# Are FGFR and IDH1-2 alterations a positive prognostic factor in intrahepatic cholangiocarcinoma? An unresolved issue

**DOI:** 10.3389/fonc.2023.1137510

**Published:** 2023-04-24

**Authors:** Giovanni Brandi, Chiara Deiana, Linda Galvani, Andrea Palloni, Angela Dalia Ricci, Alessandro Rizzo, Simona Tavolari

**Affiliations:** ^1^ Medical Oncology, IRCCS Azienda Ospedaliero-Universitaria di Bologna, Bologna, Italy; ^2^ Department of Medicine and Surgical Sciences, University of Bologna, Bologna, Italy; ^3^ Medical Oncology Unit, National Institute of Gastroenterology, “Saverio de Bellis” Research Hospital, Bari, Italy; ^4^ IRCCS Istituto Tumori “Giovanni Paolo II” of Bari, Struttura Semplice Dipartimentale di Oncologia Medica per la Presa in Carico Globale del Paziente Oncologico “Don Tonino Bello”, Bari, Italy

**Keywords:** FGFR, IDH, cholangiocarcinoma, prognosis, molecular alteration

## Abstract

Despite representing some of the most common and investigated molecular changes in intrahepatic cholangiocarcinoma (iCCA), the prognostic role of FGFR and IDH1/2 alterations still remains an open question. In this review we provide a critical analysis of available literature data regarding this topic, underlining the strengths and pitfalls of each study reported. Despite the overall poor quality of current available studies, a general trend toward a better overall survival for FGFR2 rearrangements and, possibly, for FGFR2-3 alterations can be inferred. On the other hand, the positive prognostic role of IDH1/2 mutation seems much more uncertain. In this scenario, better designed clinical trials in these subsets of iCCA patients are needed in order to get definitive conclusions on this issue.

## Introduction

Cholangiocarcinoma (CCA) encompasses a group of heterogeneous and rare tumours with poor prognosis, including intrahepatic CCA (iCCA) and extrahepatic (eCCA), with the latter further subdivided into perihilar (pCCA) and distal (dCCA) (1). Overall, these malignancies account for approximately 10-15% of primary liver cancers with an incidence on the rise counting between 0.3 – 6 cases/100’000 inhabitants in Western countries (2, 3). Unfortunately, potentially curative surgical resection is feasible in about 25% of patients at diagnosis and even following radical surgery, relapse rate remains high (4). Most patients present with locally advanced, unresectable, or metastatic disease, and palliative chemotherapy with cytotoxic drugs such as gemcitabine plus cisplatin represents the standard treatment in this setting, with an associated overall survival (OS) of 11.7 months (5). The addition of immunotherapy with the anti PD-L1 drug Durvalumab to the well-known doublet of cisplatin and gemcitabine has recently been proven to be associated with an increased OS, so a change in the first-line setting paradigm could potentially happen soon (6).

FOLFOX regimen, comprised of fluorouracil and oxaliplatin doublet, has been proven to give an advantage compared to active symptom control in second-line setting (7), and therapies directed against different target such as FGFR, IDH, NTRK, Her2 and more, are being investigated.

Large scale sequencing analyses regarding iCCA tumor biology have provided new insights, unravelling the complex and heterogeneous genomic landscape of this disease (8). Currently potential therapeutic targets have been identified in nearly 40% of biliary tract cancers, with the most promising in iCCA patients including isocitrate dehydrogenase 1 (IDH1) and fibroblast growth factor receptor 2 (FGFR2) (9). Anti-FGFR drugs such as Pemigatinib, Infigratinib and Futibatinib and anti-IDH therapies such as Ivosidenib have been approved in pre-treated patients (10, 11).

A whole genome and epigenetic analysis of CCA by Jusakul et al. lead to the definition of four distinct clusters of CCA, with the two clusters pertaining iCCA being defined by the presence of PD-1 or PD-L1 up-regulation, aberration in T- Cells transduction and CD28 co-stimulation (cluster 3), or BAP1, IDH1, IDH2, FGFR alteration, upregulation of FGFR or PI3K pathways and DNA hypermethylation (cluster 4). The study suggested that cluster 4 was associated with the best prognosis of all subtypes (p < 0.001) and, although no specific OS data on FGFR or IDH in iCCA were provided, it was enough to spark interest in the prognostic role of these mutations (12).

Despite the numerous papers that tried to address this topic, the prognostic role of FGFR2 and IDH genetic alterations in iCCA still remains a controversial issue in the medical community, with several studies reporting conflicting results and a poor quality of data.

The aim of this review is to analyze the prognostic relevance of FGFR2 and IDH alteration in iCCA, with a special focus on the data available from each paper (**Tables 1**, **2**) and their subsequent bias or shortcomings (**Tables 3**, **4**).

**Table 1:** OS in FGFR2/3 altered and OS in FGFR2 rearranged patients compared to OS in iCCA (*) or Biliary Tract Cancer BTC (#) cases.

Reference	N° of BTCs (N° of CCAs)	% advanced stage	N° of iCCA cases with FGFR2/3 alterations	OS in iCCA patients with FGFR2/3 alterations	p-value	N° of iCCAs cases with FGFR2 rearrangment/fusion	OS in FGFR2 mutated vs FGFR2 wild-type patients	p-value	Notes
Jusaku et al. Cancer Discov, 2017 (12)	489 BTCs (310 iCCAs)	For iCCA: 58% stage III and IV	15	–	–	11		–	The cluster of BTCs including FGFR altered cases had a better OS compared to other clusters, with a p < 0.001
Javle et al. Cancer, 2016 (13)	321 BTCs (224 iCCAs)	For iCCA: 74% stage III and IV	42	NA vs 43 months	0.005	30	NA vs 43 months (*)	0.001	
Churi et al. PLOS ONE. 2014 (14)	75 BTCs (55 iCCAs)	For iCCA cases: 62% stage IV, 31% stage III	7	NA vs 17.6 months	0.00973	3	–	–	
Graham et al. Hum Pathol, 2014 (15)	156 BTCs (96 iCCAs)	–	–	–	–	12	123 vs 37 (#) months	0.039	OS data are compared to all BTCs and not only to iCCAs
Jain et al. JCO Precis Oncol, 2018 (16)	377 BTCs (273 iCCAs)	71.3 % stage III or IV	95 (including FGFR1-2-3-4 and FGF 19 alterations)	37 vs 20 (#) months	< 0.001	60	–	–	No difference in OS for FGFR alterations vs FGFR2 fusions was found (33 vs 37 months; p = 0.657)
Rizzato et al. Eur J Cancer, 2022 (17)	286 BTCs (183 iCCAs)	70.6 % stage III-IV (72.1 % in iCCA cases)	19/127 analyzed	24.9 vs 14.8 months (*)	0.02	13	29.2 vs 15.0 months (*)	0.03	
Maruki et al. J Gastroenterol, 2021 (18)	445 BTCs (272 iCCAs)	52% stage IV, 26% recurrence, 22% locally advanced	–	–	–	20	38.8 months vs unknown	–	OS data are on 18 treated FGFR2 BTC patients (defined as the time from the start of first-line chemotherapy). No OS data for FGFR2 wild-type patients was provided.
Abou-Alfa et al. Target Oncol, 2022 (19)	132 iCCAs	22.7% stage III, 53.8 stage IV	17	–	–	15	31.3 vs 21.7 months	–	In the OS analysis only 9 patients with FGFR2 fusions and only 109 wild-type patients were included
Boerner et al. Hepatology, 2021 (20)	412 iCCAs	43% stage IV	–	–	–	47	46.8 vs 29.6 months	0.20	
Arai et al. Hepatology. 2014 (21)	102 BTCs (66 iCCAs)	41% stage III, 34% stage IV	–	–	–	9	Data not provided, positive trend (*)	0.616	
Nepal et al. Hepatology. 2018 (22)	150 iCCAs	18% stage III, 20.7+18% stage IVa+b	–	–	–	18	Data not provided, negative trend	0.0001	Of the 150 iCCA cases, only 122 were analyzed for FGFR2 fusions.

OS is defined in this table as the time from diagnosis to last contact/death, if not otherwise specified in the notes. % of advance stage refer to all BTC analyzed, if not otherwise specified.

**Table 2:** OS in IDH1/2 mutated and OS in IDH1 mutated patients compared to OS in iCCA (*) or Biliary Tract Cancer BTC (#) cases.

Reference	N° of BTCs (N° iCCAs)	% advanced disease	N° of iCCA cases with mIDH1/2	OS in mIDH1/2 vs wt iCCA patients	p-value	N° of iCCA cases with mIDH1	OS in mIDH1 vs wild-type iCCA	p-value	Notes
Javle et al. Cancer, 2016 (13)	321 BTCs (224 iCCAs)	For iCCA: 74% stage III and IV	–	–	–	40	47.18 *vs* 47.40 months	0.56	
Churi et al. PLos One, 2014 (14)	75 BTC (55 iCCAs)	For iCCA: 62% stage IV, 31% stage III	–	–	–	13	–	–	No prognostic significance has been reported for IDH1 alterations
Rizzato et al. Eur J Cancer, 2022 (17)	286 BTCs (183 iCCAs)	70.6 % stage III-IV (72.1 % in iCCA cases)	34/127 analysed	25.9 vs 16.3	0.06	24/127 analysed	–	–	
Boerner et al. Hepatology, 2021 (20)	412 iCCAs	43% stage IV	102	OS 36.1 vs 29.6	0.08	84	–	–	A trend toward increased OS was observed in patients with mIDH1/2
Ma et al. BMC Cancer, 2020 (23)	130 iCCAs	–	21	OS not provided, but a positive trend at univariate analysis is reported	0.031	–	–	–	OS not provided. Multivariate analysis confirmed a positive trend for OS (p = 0.038)
Wang et al. Oncogene, 2013 (24)	326 iCCAs	–	34	OS not provided, but a positive trend is reported	0.028	22	OS not provided, but a positive trend is reported	0.169	A better OS for mIDH2 (p=0.05) than mIDH1 (p = 0.169) is reported
Zhu et al. Ann Surg Oncol, 2014 (25)	200 iCCAs	–	40	31.25 vs 30.95 months	0.96	31	39.3 vs 30.95 months	0.94	OS mIDH2 vs wild-type: 25.3 vs 30.95 months (p = 0.75)
Ruzzenente et al. Ann Surg Oncol, 2016 (26)	91 BTCs 35 iCCAs	For iCCA: 20% stage III and 20% stage IV	7	Data not provided	–	6	–	–	
Goyal et al. Oncologist, 2015 (27)	104 iCCAs	100% stage III-IV	30	15.0 vs 20.1 months	0.17	26	–	–	

OS is defined in this table as the time from diagnosis to last contact/death, if not otherwise specified in the notes. % of advance stage refer to all BTC analyzed, if not otherwise specified. NA or -: data Not Available.

**Table 3:** Trial’s bias/shortcoming, if relevant data are provided, a checkmark sign is used (**✓**), otherwise a (NA) standing for Not Available is used.

Reference	iCCA data only	Appropriate sample size for patients with FGFR alterations	OS patients with FGFR alterations	OS FGFR wild-type patients	Stage	Surgery	Chemotherapy	Targeted therapy
Jusakul, Cancer Discov, 2017 (12)	NA	NA	NA	NA	✓	NA	NA	NA
Javle, Cancer, 2016 (13)	✓	✓	✓ (1)	✓ (1)	✓	✓	✓	✓
Churi, PLOS ONE. 2014 (14)	✓	NA	✓ (1)	✓ (1)	✓	✓	✓	✓
Graham,Hum Pathol, 2014 (15)	NA	NA	✓ (1)	✓ (1)	NA	✓	NA	NA
Jain, JCO Precis Oncol, 2018 (16)	NA	✓	✓ (1)	✓ (1)	✓	✓	✓	✓
Rizzato, Eur J Cancer, 2022 (17)	✓	NA	✓ (2)	✓ (2)	✓	✓	✓	✓
Maruki, J Gastroenterol, 2021 (18)	✓	✓/NA *	✓ (2)	NA	✓	NA	✓	✓
Abou-Alfa, Target Oncol, 2022 (19)	✓	NA	✓ (1)	✓ (1)	✓	✓	✓	✓
Boerner, Hepatology, 2021 (20)	✓	✓	✓ (1)	✓ (1)	✓	✓	✓	✓
Arai, Hepatology. 2014 (21)	✓	NA	NA	NA	✓	NA	NA	NA
Nepal, Hepatology. 2018 (22)	✓	NA	NA	NA	✓	✓	NA	NA

*Although patients with FGFR alterations were 20, only 18 were included in OS analysis. Appropriate size sample was arbitrarily chosen as ≥ 20 patients. OS data could refer from initial diagnosis (1) to time of last contact/death or time from the start of systemic first-line of treatment (2) to last contact/death.

**Table 4:** Trial’s bias/shortcoming, if relevant data are provided, a checkmark sign is used (✓), otherwise a (NA) standing for Not Available is used.

Paper	iCCA data only	Appropriate size sample for IDH1/2 mut patients	OS for IDH1/2 mut patients	OS for IDH1/2 wild-type patients	Disease stage provided	Surgery	Chemotherapy	Target therapy
Javle et al. Cancer, 2016 (13)	✓	✓	✓ (1)	✓ (1)	✓	✓	✓	✓
Churi et al. PLos One, 2014 (14)	✓	NA	NA (1)	NA (1)	✓	✓	✓	✓
Rizzato et al. Eur J Cancer, 2022 (17)	✓	✓	✓ (2)	✓ (2)	✓	**✓**	✓	✓
Boerner et al. Hepatology, 2021 (20)	✓	✓	✓ (1)	✓ (1)	✓	✓	✓	NA
Ma et al. BMC Cancer, 2020 (23)	✓	✓	✓ (1)	✓ (1)	NA	✓	NA	NA
Wang et al. Oncogene, 2013 (24)	✓	✓	NA (1)	NA (1)	NA	✓	NA	NA
Zhuet al. Ann Surg Oncol, 2014 (25)	✓	NA	✓ (1)	✓ (1)	✓	✓	NA	NA
Ruzzenente et al. Ann Surg Oncol, 2016 (26)	NA	NA	NA (1)	NA (1)	✓	✓	✓ (adjuvant)	✓
Goyal et al. Oncologist, 2015 (27)	✓	✓	✓ (2)	✓ (2)	✓	✓	NA	NA

Appropriate size sample was arbitrarily chosen as ≥ 20 patients. OS data could refer from initial diagnosis (1) to time of last contact/death or time from the start of systemic first-line treatment (2).

## Fibroblast growth factor receptor 2 (FGFR2)

Fibroblast growth factor receptors (FGFRs) 1-4 are transmembrane tyrosine kinase receptors that, when bound to their ligand FGF, are responsible for the activation of pathways such as RAS/RAF/MAPK and PI3K/AKT, involved in proliferation, migration and anti-apoptotic signals (28).

FGFR2 rearrangements or fusions are the most frequent molecular events detected in iCCA, occurring in a proportion of patients ranging from 3 to 15% of all iCCA cases, whereas mutations and amplifications seem to be rarer (29). The majority of these abnormalities results in a gain-of-function of the receptor, that in turn activates multiple downstream oncogenic signaling pathways (30).

From their first discovery and identification, FGFR2 fusions have been suggested to define a unique clinical iCCA subtype associated with a younger age at onset, female sex, earlier stage and a more indolent disease progression (13, 31). Initial prognostic data on alteration of the FGF pathway and specifically on FGFR2 fusions showed a trend toward better OS data (13, 14).

In a landmark study by Graham and colleagues, the authors reported 13 cases of FGFR2 translocations among 156 surgical cases of biliary tract tumors, with almost all altered cases being iCCAs (12); a significantly longer survival was observed in patients with FGFR2 translocations compared to wild-type ones, with a median OS of 123 vs 37 months, respectively (p = 0.039) (15). Furthermore, of the 12 cases of FGFR2 altered iCCAs, only 3 patients developed recurrence or secondary lesions, all with a statistically longer disease-free interval compared to the FGFR wild-types patients (p = 0.007) (15). It should be noted however that relevant prognostic information, such as stage before resection or systemic treatment, were not provided.

An interesting study by Buckarma analyzed OS for resected iCCA with FGFR2 fusions (12 patients) vs wild-type resected patients (87 patients) and found out that 5 years OS was far longer for altered patients (83 vs 32%, p = 0.01) (32).

Another remarkable study was the 2016 paper by Javle et al. (13) as it provided data on patients’ characteristics, treatment received, and it differentiated well between different biliary tract cancer subtypes. At the univariate analysis of the iCCA subset (224 cases, 74% stage III or IV), the 30 FGFR2 rearranged cases had a strong survival advantage (OS NA vs 43 months, p = 0.001) and this positive prognostic correlation with FGFR alteration was confirmed at the multivariate analysis (p = 0.03). However, it was not specified whether with the citation “FGFR alteration”, the authors referred only to FGFR2 fusions or to all FGFR alterations (42 cases in total) found in the iCCA samples.

A favorable prognostic role of FGFR genetic aberrations has been also suggested by another study on 377 CCAs (16). In this cohort of patients, 95 CCAs harbored FGFR genetic aberrations, with most of them being FGFR2 fusions (63 cases), and almost all occurring in iCCAs (60 cases, 95.2%). Patients with FGFR genomic aberrations experienced a longer OS compared to wild-type patients (37 vs 20 months, respectively; p < 0.001) and this advantage was preserved even when considering only stage III or IV patients (24 v 17 months; p < 0.004). However, a pitfall of this study is that no specific OS data on FGFR2 fusions in iCCA were presented, with data from all biliary tract cancer being included.

Of note, in this paper a longer OS was also observed after excluding those patients treated with FGFR inhibitors, showing an OS of 30 in FGFR altered patients vs 20 months in FGFR wild type patients; p = 0.0266) (16).

In fact, it should be noted that the introduction of new lines of therapy with the use of specific anti-FGFR drugs in FGFR altered patients can be a strong confounding factor, as it raises the question of whether the gain in OS seen in some trial could be due to the intrinsic positive prognostic role of FGFR alteration or if it could merely be due to the predictive role to FGFR target therapy.

Several other papers tried to answer this question in their trials. For example, in the BITCOIN trial (17), out of 286 biliary tract cancer cases analyzed, the 24 patients (8.4%) with FGFR2/3 alteration had a better OS compared to FGFR wild-type cases (29.2 vs 14.4 months, p = 0.003). Focusing on the 183 iCCAs, the BITCOIN trial provided OS data from the time of the start of first-line therapy in patients with FGFR2-fusions (13 patients, with 11 undergoing 1st line treatment, OS 29.2 vs 15.0 months, p = 0.03) and in patients with all type pf FGFR2/3 alterations (19 cases, with 17 included in the analysis, OS 24.9 vs 14.8 months, p = 0.02); notably, the survival advantage held true even when censoring the 8 cases who received FGFR targeted therapies (HR = 0.32, 95% CI 0.12–0.85, p = 0.02).

On the other hand, the Japanese PRELUDE study (18) suggests that the OS advantage could be due to targeted therapies. Out of 20 FGFR2-rearranged patients with recurrent or advanced iCCA, 18 underwent first-line chemotherapy (cisplatin/gemcitabine or cisplatin/S-1) with a progression-free survival (PFS) in line with known data (8.9 months compared to the 8.0 months of the ABC-02 trial (5); however, 13 of them had subsequent anti-FGFR2 therapies, obtaining an OS of 38.8 months. It should be noted that the study did not provide PFS or OS data of either FGFR wild-type patients or of those patients with FGFR alterations who did not receive targeted therapy, thus no solid conclusions can be drawn.

What seems clear from the published data is that FGFR alterations are not associated with a different response to first-line chemotherapy compared to FGFR wild-type patients.

A retrospective study (19) on the effect of first-line chemotherapy on FGFR2 altered patient showed that, although OS was longer for altered patients (31.3 vs 21.7 months), this was not due to a better response to first-line treatment (93% of cases platinum based) with a PFS of 6.2 months for altered cases vs 7.2 months for wild-type cases. Interestingly, even after excluding patients who had received an FGFR inhibitor, a slightly longer PFS for second-line treatment was observed (5.6 months for 8 mutated patients vs 3.7 months for 81 wild-type cases). It should be noted however that size sample was small and that only descriptive statistics was employed (p-values were not provided) (19).

Conversely, not all evidences support the hypothesis of a positive prognostic correlation in patients with FGFR2 alterations.

A recent paper in 2021 failed to find a significant correlation between the presence of FGFR2 fusion gene and prolonged OS. Considering a large number of iCCAs (412), both resected and not resected, although numerically the OS was longer for the 47 FGFR2 mutated patients (46.8 vs 29.6 months), it was not statistically relevant (p = 0.20) (20).

Similarly, an Asian cohort study reported no statistically significant survival differences in FGFR2 fusion-positive patients, although the examined cohort was small (9 patients out of 66 iCCAs) and no treatment data was provided (21). Interestingly, another paper on 18 FGFR2 fusion cases out of 122 analyzed resected iCCA cases, reported a trend toward a worse OS (p < 0.0001), however, neither precise data on treatment and stage disease for mutated patients, nor precise OS for wild-type and mutated patients were provided (22).

### Isocitrate dehydrogenase (IDH)

Isocitrate Dehydrogenase (IDH) 1 and IDH2, residing respectively in the cellular cytoplasm and mitochondria, are NADP+-dependent enzymes which catalyze the oxidative decarboxylation of isocitrate to α-ketoglutarate (α-KG) (33). Somatic mutations in IDH1 gene result in the production of a neomorphic enzyme that leads to the synthesis of the 2-Hydroxyglutarate (D-2HG) oncometabolite, instead of α-KG, thus altering numerous processes involved in epigenetics and cellular metabolism, and favoring tumor maintenance through immune evasion (34–37). Indeed, due to its structural similarity with α-KG, D-2HG is able to inhibit DNA and histone demethylases, inducing a hypermethylated phenotype in target cells and the silencing of genes involved in cell differentiation and immune response (37). Moreover, D-2HG may induce mitochondrial dysfunction, because of succinate dehydrogenase (SDH) inhibition, that results in intracellular succinate increase in levels and mitochondrial respiration inhibition (37).

The most frequent mutations of the IDH1 gene are single amino acid changes that occur in the catalytic site, especially at arginine 132 residue, leading to a gain of function of the enzyme (38). The presence of mutated IDH1 among CCA patients is higher in those affected by iCCA compared with those with eCCA, with an overall frequency of 13.1% vs 0.8% respectively (39). The presence of circulating 2-HG has been hypothesized as an alternative biomarker of IDH1/2 mutational status in CCA, and its levels seems to correlate with tumor burden (40). Recently, the ClarIDHy trial, a phase 3 multicenter randomized study, tested the efficacy of the IDH1 inhibitor Ivosidenib in patients with pretreated, non resectable or metastatic CCA with IDH1 mutation, observing an improvement in PFS compared to placebo (mOS 2.7 months vs 1.4 months respectively) (11). This finding represents a new hope in this particularly challenging disease. Despite the efforts made to understand the prognostic significance of IDH1/2 mutations (mIDH1/2) in CCA patients, no clear evidence has been established so far.

Focusing on patients with resected disease, in a work by Ma et al. on 130 resected iCCA patients, the presence of mIDH1/2 showed a significant gain in DFS and OS, showing a trend toward increased OS (p = 0.031 at univariate analysis and 0.038 at multivariate analysis) (23). Another interesting work by Wang et al. (24) on 326 resected iCCA cases, from a Chinese and a US cohort, discovered mIDH1/2 to be associated with longer OS (p = 0.028) and with a longer time to tumor recurrence after resection in the multivariate analysis (p 0.021). Probabilities of tumor recurrence after resection at 1, 4 and 7 years in patients with mIDH1/2 iCCA (10.5%, 45.3% and 45.3%, respectively) were significantly lower than those with wild-type IDH1 or IDH2 (41.7%, 71.5% and 81.3%, respectively). Nonetheless, many important features, such as tumor stage and further treatment strategies, were not provided.

Zhu et al. conducted a very articulate analysis of genomic profiling of 200 iCCA cases, considering data about mIDH1 and mIDH2 patients separately and together, but it did not find a significant association between mIDH1/2 and OS when compared to wild type patients (mOS 31.25 vs 30.95 months respectively, p = 0.96). All the patients included in this study underwent surgical resection, but no information about other subgroups of patients with advanced, non resectable iCCAs were provided (25).

The same result was reached by Ruzzenente et al. (26) in a study on resected iCCA patients. The paper reported no evident influence of mIDH1 on OS in resected iCCA patients; however, despite thorough details regarding differentiation between CCA subtypes and molecular features, no precise data and p-values about survival in iCCA mIDH1/2 patients were provided.

The following studies, on the other hand, aimed to assess the prognostic role of mIDH1/IDH2 in more heterogenous populations, considering patients with both resected and unresectable/advanced disease.

A recent work by Rizzato et al. claimed at the multivariate model a correlation between mIDH1/2 and improved survival in a population of iCCA patients (17), reporting a median OS of 25.9 months compared to 16.3 months in wild-type patients (p = 0.06). In this paper, the benefit appears independent from targeted therapy administration, since no mutated IDH1/2 patients had received IDH1 inhibitors.

In contrast, the previously mentioned work by Javle et al., characterized by a very solid design with relevant features reported, found no prognostic relevance of the presence of mIDH1/2 in iCCA (47.4 vs 47.1 months, p = 0.56) (13).

Consistent with these results, the work by Churi et al. suggested no correlation between mIDH1 and OS (14). Several important characteristics were provided in the paper, such as disease stage and administrated treatments, but it should be noted that the mutated IDH1/2 size simple was very small (13 patients) and p-values and OS are lacking.

Goyal et al. assessed prognostic features of advanced iCCA patients, again reporting no statistically significant association between mIDH1/2 and OS (15.0 vs 20.1 months, p = 0.17), defined both as the time from diagnosis of primary unresectable or metastatic disease and the time from initial diagnosis in the 30 patients diagnosed as early-stage disease who then experienced a recurrence afterwards (27).

Finally, in the previously mentioned work by Boerner et al. focused exclusively on iCCA patients, no statistically significant differences in OS were observed in mIDH1/2 patients, although the presence of the mutation was correlated with a trend toward an improved OS (p = 0.08) (20). Furthermore, this study investigated separately the role of mIDH1 and mIDH2, finding again no association with patients’ outcome.

## Discussion

Several confounding factors should be noted when trying to interpret available data from clinical trials on the prognostic role of FGFR or IDH1/2 molecular alterations.

First of all, most of the trials regarding FGFR included both resected and not resected cases and usually OS data according to stage for mutated patients were not provided. This is of course understandable, given the rarity of the mutations and the subsequent small sample size of the clinical trials (with only 3 studies including more than 20 FGFR altered cases); however, it is worth noting that even the trials with more cases failed to provide this important data. Some findings seem to suggest that FGFR mutated patients are more likely to form a mass-forming cancer, making the surgical approach easier for this subset of CCAs (16). If this was true, it would raise the question of whether the OS advantage observed in FGFR mutated patients could be due to the greater number of patients who underwent resection and not to an intrinsic characteristic of these patients. Thus, separate data set on resected patients vs patients undergoing systemic treatment should be made available.

Regarding the prognostic values of IDH mutations on resected iCCA patients specifically, four studies focused on this subset of patients, but results are heterogeneous, with some paper pointing towards a benefit in OS and DFS, while others denying such benefit. As with FGFR mutated cases however, no OS data according to stage disease were provided across all trials.

Another important question is to rule out whether the presence of FGFR alteration could affect response to systemic therapies. Several trials agree that there is not impact on response to first-line chemotherapy (18, 19). However, this is less clear for further lines of treatment, with one trial suggesting a slightly longer PFS for second-line chemotherapy, although with the strong bias of a retrospective descriptive analysis only (19). Regarding the effect of FGFR target therapies on OS, two trials confirmed the positive prognostic role of FGFR alterations on their subset of patients, even after censoring those who had received targeted drugs (16, 17), thus suggesting that the prognostic role of these molecular changes could be independent to the administered targeted treatments. On the other hand, one trial suggested the opposite (18), ascribing the OS gain to anti-FGFR therapies, although it did not provide OS for FGFR patients who had not received target therapies, making it impossible to draw definitive conclusions. Regarding IDH mutation, data are even scarcer but seeing as no patients received IDH target paper in the mentioned trials, there is no risk of this type of treatment being a confounding factor.

In addition, a certain inconsistency on data provided furtherly complicates the matter, namely: a) data often refers to all biliary tract cancers and not specifically to iCCA; b) several trials do not distinguish between FGFR2 rearrangements and mutations or between IDH1 and IDH2; c) the definition of OS in not always unanimous, as the starting point can refer to the initial diagnosis in some trials and to the start of first-line chemotherapy in others.

Finally, it should be noted that in the present review we did not consider several other known prognostic factors for iCCA, including the clinical characteristics of the patients (performance status, age, years of diagnosis, presence of hepatic diseases such as hepatolithiasis or cirrhosis) and the histological features of tumor (size, grade, presence of macroscopic vascular invasion, positive resection margins, large duct vs small duct iCCA), as the quality of these data was already poor. Furthermore, another important issue that must be carefully considered when evaluating the possible prognostic role of IDH and FGFR molecular alterations in iCCA is the co-occurrence of other mutations that could potentially influence patients’ survival. In the majority of genomic studies, FGFR2 and IDH alterations tend to be mutually exclusive and to cluster with BAP1 mutations in a subgroup of patients that is distinct from the subgroup of patients carrying TP53, KRAS, SMAD4 and ARID1A genomic alterations (9, 12, 41). Of note, while co-occurrence of IDH/BAP1 or FGFR2-fusion/BAP1 mutations is almost exclusively found in small bile duct iCCA and associates with good prognosis, KRAS, TP53 and SMAD4 mutations are frequently observed in large bile duct iCCA and associate with dismal prognosis (41). The association of FGFR2 alterations with KRAS or TP53 mutations has been also reported in iCCA patients, although with a very low frequency (42). These molecular findings suggest that combined therapeutic strategies targeting these oncogenic drivers could improve the clinical outcome in selected iCCA patients carrying these genetic alterations, deserving further investigation in future clinical studies.

All these above-mentioned prognostic factors should be rendered available and analyzed when assessing the possible prognostic role of FGFR and IDH genetic alterations in iCCA; however, due to poor quality of available data, it is difficult to draw definitive conclusions on this issue from current studies. A general trend toward a better OS for FGFR2 rearrangements and, possibly, for FGFR2-3 alterations could be inferred, likely independent from the treatment provided. However, the presence of few trials reporting no statistical relevance (or even a worse OS) should encourage larger clinical trials to address this issue. Regarding IDH1-2 mutations, on the other hand, only a handful of papers describe a positive prognostic role for these alterations, with most of the paper leaning toward no prognostic correlation.

As the presence of more small retrospective trials is unlikely to resolve the issue, we encourage the creation of accurate and well-designed prospective trials, with a good sample size including only iCCA, with a clear study population in mind (resected vs non resected, advanced vs early stage, FGFR2 translocations vs all FGFR mutations), and in which all confounding prognostic factors are described and taken into account at multivariate analysis, such as clinical characteristics, tumor histopathological features, coexisting mutations and treatment provided (**Table 5**). Only when all these parameters are available and valuable the prognostic relevance of IDH and FGFR molecular alterations in iCCA can emerge and be clarified.

**Table 5:** **“**Ideal” parameters to be included in a trial aimed at defining the prognostic role of FGFR or IDH mutations in iCCA.

	Characteristics of a well-designed trial	Characteristics to avoid in a trial
**Type of trial**	Prospective multicenter trials	Retrospective single center
**Types of cancer included**	Data on iCCA patients only	No distinction between data on iCCA, eCCA and gallbladder cancer
**Number of patients**	Good sample size	Small sample size
**Statistical analysis**	Complete statistical analysis included in the final report	The trial reports only trends, without specific data
**Analyzed populations**	Population well separated according to mutation For example: - FGFR2 translocation vs wild type - IDH1 vs wild type	No clear distinction between populations and relative mutations For example: - all FGFR mutations vs wild type - IDH1-2 vs wild type
**Stages**	Trial includes only early or advances stages. In both cases the percentage of patients with a certain stage is specified	All stages are included in the trial. No specific percentages on how many patients falls within a certain stage are provided
**Surgery**	Should include only unresected or resected patients. Data on whether the resection was radical or whether the margin were free of neoplasia is provided	Both resected and unresected patients are included; No data on how many patients underwent surgery is available
**Treatment**	The type and number of chemotherapy regiment provided is specified	No data on treatment is available
**Target therapy**	Patients receiving target therapy against the examined mutation are excluded from analysis	All patients are included in the analysis, even those receiving target therapy.
**Coexisting mutations**	Data on coexisting mutations is provided and their presence is considered in the statistical analysis	The presence of coexisting mutations is not reported
**Clinical characteristics**	Data on median age, performance status, presence of cirrhosis or other major comorbidities are provided and considered at the multivariate analysis	No clinical characteristics are provided
**Histological features**	Data on presence of macroscopic vascular invasion, large duct vs small duct iCCA, positive resection margins, are provided and considered at the multivariate analysis	No data on histological features are provided.

iCCA, intrahepatic cholangiocarcinoma; eCCA, extrahepatic cholangiocarcinoma.

Treatment paradigm of iCCA is progressively shifting towards precision oncology; in this scenario, a better understanding of the prognostic role of genetic aberrations driving iCCA carcinogenesis appears mandatory to improve the clinical outcome of these patients.

## Author contributions

GB and ST: conception of the study, data interpretation and drafting of the article; CD, LG, AP: drafting of the article; AR and ADR: critical revision of the manuscript. All authors contributed to the article and approved the submitted version.
